# Water-Pipe Smoking Exposure Deregulates a Set of Genes Associated with Human Head and Neck Cancer Development and Prognosis

**DOI:** 10.3390/toxics8030073

**Published:** 2020-09-18

**Authors:** Vanessa M. López-Ozuna, Ishita Gupta, Ryan Liu Chen Kiow, Emad Matanes, Hadeel Kheraldine, Amber Yasmeen, Ashraf Khalil, Semir Vranic, Ala-Eddin Al Moustafa, Halema F Al Farsi

**Affiliations:** 1 Segal Cancer Center, Lady Davis Institute of Medical Research, JGH, McGill University, Montreal, QC H3T IE2, Canada; vanessa.lopez2@mail.mcgill.ca (V.M.L.-O.); ryan.kiow@gmail.com (R.L.C.K.); emad.matanes@gmail.com (E.M.); amber.yasmeen@mail.mcgill.ca (A.Y.); 2 College of Medicine, QU Health, Qatar University, Doha PO Box 2713, Qatar; ishugupta28@gmail.com (I.G.); hk1805332@student.qu.edu.qa (H.K.); svranic@qu.edu.qa (S.V.); aalmoustafa@qu.edu.qa (A.-E.A.M.); 3 Biomedical Research Centre, Qatar University, Doha PO Box 2713, Qatar; 4 College of Pharmacy, QU Health, Qatar University, Doha PO Box 2713, Qatar; akhalil@qu.edu.qa

**Keywords:** smoke, water pipe, head and neck cancers, gene dysregulation, oral epithelial cells

## Abstract

Water-pipe smoking (WPS) is becoming the most popular form of tobacco use among the youth, especially in the Middle East, replacing cigarettes rapidly and becoming a major risk of tobacco addiction worldwide. Smoke from WPS contains similar toxins as those present in cigarette smoke and is linked directly with different types of cancers including lung and head and neck (HN) carcinomas. However, the underlying molecular pathways and/or target genes responsible for the carcinogenic process are still unknown. In this study, human normal oral epithelial (HNOE) cells, NanoString PanCancer Pathways panel of 770 gene transcripts and quantitative real-time polymerase chain reaction (qRT-PCR) analysis were applied to discover differentially expressed genes (DEG) modulated by WPS. In silico analysis was performed to analyze the impact of these genes in HN cancer patient’s biology and outcome. We found that WPS can induce the epithelial–mesenchymal transition (EMT: hallmark of cancer progression) of HNOE cells. More significantly, our analysis of NanoString revealed 23 genes deregulated under the effect of WPS, responsible for the modulation of cell cycle, proliferation, migration/invasion, apoptosis, signal transduction, and inflammatory response. Further analysis was performed using qRT-PCR of HNOE WPS-exposed and unexposed cells supported the reliability of our NanoString data. Moreover, we demonstrate those DEG to be upregulated in cancer compared with normal tissue. Using the Kaplan–Meier analysis, we observed a significant association between WPS-deregulated genes and relapse-free survival/overall survival in HN cancer patients. Our findings imply that WPS can modulate EMT as well as a set of genes that are directly involved in human HN carcinogenesis, thereby affecting HN cancer patients’ survival.

## 1. Introduction

Tobacco smoking is the most common preventable risk factor for several non-communicable diseases, such as, cardiovascular, lung, diabetes as well as cancer, and can be considered lethal [1,2]. Widespread tobacco consumption is somewhat attributed to the variation in available consumption methods, such as cigarettes, electronic cigarettes (E-cigarettes), cigars, and water-pipe smoking (WPS). Recently, global trend of tobacco smoking has started to shift towards WPS in addition to E-cigarettes [3,4,5] with approximately 100 million smokers using WPS on daily basis [6], leading to nearly 5 million deaths annually [7]. Global increase in WPS use is due to several factors including its availability in several delectable flavors and aromas along with its association with socializing, relaxation, and entertainment. Additional motives include peer-pressure, low-cost, fashion, and inquisitiveness [3,8]. Interestingly, people from the Middle East and those of Middle Eastern descent in Western countries smoke WPS, as they consider it to be a part of their culture, thus giving rise to this trend in the Western world [9].

Smoke emanating from WPS includes toxins resembling those found in cigarettes, such as carbon monoxide (CO), hydrocarbons, and carcinogenic polycyclic aromatic volatile aldehydes [10,11]. In comparison to a cigarette, which accounts for 500–600 mL of smoke inhalation per unit, a single WPS session accounts for approximately 90,000 mL of smoke inhalation; making WPS 4-times higher in CO exposure and 56-times higher in inhaled smoke volume [11]. Additionally, it has been pointed out that nicotine concentration in plasma of individuals smoking one WPS daily is similar to those smoking 10 cigarettes a day [12,13]. Out of 300 chemical compounds, which have been identified in inhaled WPS smoke, 82 have been labelled as “toxicants” [14,15] including polyaromatic hydrocarbons, heterocyclic compounds, carbonylic compounds and volatile organic compounds, tar, nicotine, carbon monoxide, nitrosamines, heavy metals, metal nanoparticles, phenolic compounds, flavoring chemicals (base propylene glycol, glycerol, vanillin, cinnamaldehyde), and free radicals which can induce head and neck (HN) as well as pulmonary toxicity [15,16]. Nevertheless, popular belief considers WPS less harmful than cigarette smoking. However, research shows that both methods of tobacco consumption lead to serious health problems including a variety of oral and systemic diseases, such as periodontal affliction, cardiovascular, and pulmonary disorders [17,18,19,20,21]. On the other hand, we have previously reported that WPS can exhibit a substantial embryotoxicity on the early stage of the normal development [22].

To date, various studies have confirmed the association between WPS and several types of human cancers, including lung, esophageal, oral, and pancreatic carcinomas [23,24,25]. Chronic human exposure to WPS smoke alters the expression of genes involved in detoxification, xenobiotic metabolism, as well as DNA stability and repair processes, hence, increasing a susceptibility to various cancers [23,26]. We also recently demonstrated that WPS exposure can induce epithelial-mesenchymal transition (EMT) and enhance a cell invasion ability of human breast cancer cells via the activation of Erk1/Erk2 pathways [27]. However, the exact role of WPS exposure on human cancer initiation, including HN, is still unclear. Therefore, in the current study, we aimed to explore the role of chronic exposure to WPS on molecular pathways and gene targets in human normal oral epithelial cells, which can increase their susceptibility to cancer.

## 2. Materials and Methods

### 2.1. Smoking Machine Protocol and WPS Preparation

A standard smoking protocol (Aleppo Method) was used as described previously by our group [22,27]. The water pipe was prepared by padding the head with 10 gr of brand tobacco mixture known as “Two Apples”, covering it with aluminum foil and perforating the foil to allow air passage. A charcoal, “Tree Kings” brand, quick-light briquette was ignited and placed on top of the head at the beginning of the smoking session. The condensate (smoking) was collected using regular laboratory filter paper. Filters were dried and weighed before and after collecting smoke. Subsequently, smoked filters were solved in phosphate buffer saline (PBS) or keratinocyte serum-free media (KSFM) (Life Technologies, Burlington, ON, Canada) with final concentration of 20 mg/mL of smoking particles; followed by filtering PBS or KSFM solutions using 0.45μm filters (Costar, Washington, DC, USA).

### 2.2. Cell Lines

Two human normal oral epithelial (NOE) cell lines established in our laboratory [28] were used and maintained at 37 °C in a humidified atmosphere of 5% CO_2_ in air. The cells were cultured in KSFM with 5  mg/100 mL of bovine pituitary extract (BPE) (Life Technologies, Burlington, ON, Canada), and 100 µg/mL penicillin–streptomycin. Cells were treated either with 100 μg/mL of WPS in PBS or KSFM solution for 48 h.

### 2.3. RNA Extraction and Reverse Transcriptase Real-Time PCR

Total RNA was extracted from cells using RNeasy Mini Kit spin columns (Qiagen). First strand cDNA was synthesized using 5X All-In-On MasterMix (MasterMix-LR, Diamed) per manufacturer’s protocol. Reverse transcriptase real-time polymerase chain reaction (RT-PCR) was carried out on 96-well plates using iTaq Universal SYBR Green Supermix (BioRad). Concentrations for each sample were measured using the NanoDrop ND-100 spectrophotometer 119 (NanoDrop Technologies, Wilmington, DE, USA) and Qubit (Thermo Fischer Scientific, 120 Waltham, MA, USA). The primer sequences were designed using Primer ExpressTM Software v3.0.1 (ThermoFisher Scientific, Franklin, MA, USA) (Table 1).

### 2.4. NanoString

Gene expression was assessed using the NanoString PanCancer Pathway Panel (NanoString Technologies, Seattle, WA, USA) consisting of probes for 770 genes implicated in carcinogenic pathways, curated from The Cancer Genome Atlas (TCGA) data. All RCC files (direct outputs/raw data from NanoString runs) were normalized using nSolver analysis software (NanoString Technologies, Seattle, WA, USA) according to the manufacturer’s protocols (nSolver User Manual). In brief, a normalization factor was calculated by obtaining the geometric mean of the controls used for each sample and applied to the raw counts of the nCounter output data to eliminate variability that was unrelated to the samples. The resulting data were normalized again with the geometric mean of the housekeeping genes. Normalized data were log2-transformed and exported to Microsoft Excel for analysis.

### 2.5. Gene Profile and In Silica Analyses

The in silico approach used in our study helped us to support and confirm our findings (the differentially expressed genes (DEGs) were discovered by NanoString analysis as previously described). The large, publicly available database Oncomine TM consists of approximately 65 gene expression datasets and was used to explore the differential expression of our genes to compare HN cancer with respective normal tissues as well as clinico-pathological parameters. From this database we used Toruner, Ginos, Cromer, Ye, Peng, Sengupta, Estilo, Kuriakose, and TCGA datasets to evaluate mRNA expression of the discovered DEGs in normal versus malignant patient samples. In addition, TCGA Head and Neck dataset (270 patients) was used to evaluate the differences in the DNA copy numbers between smoker head and neck cancer patients compared with non-smokers with head and neck cancer. In brief, the parameters were set and the program generated the expression levels per dataset; analysis was performed, and we finally selected genes that were statistically relevant to our study. Moreover, we used a cohort of 500 HN squamous cell carcinomas (HNSCC) samples from the Pan-cancer RNA-seq dataset of the Kaplan–Meier plotter database to analyze the patients’ clinical outcome.

### 2.6. Network and Pathway Interaction

The Search Tool for the Retrieval of Interacting Genes (STRING v9.1) (https://string-db.org/) tool was used to investigate the network and interaction between the different WPS deregulated genes as well as biological function. This is a biological database and web resource of known and predicted protein–protein interactions. Briefly, we uploaded the obtained gene list and the software imported protein association knowledge from databases of physical interaction and databases of curated biological pathway knowledge; the program utilizes computational predictions to generate maps and connections between different proteins. We used this tool to highlight the importance of the potential connectivity network of our genes that need to be considered for the full understanding of the biological phenomena.

### 2.7. Statistical Analysis

In vitro assays were all performed in triplicates of at least three independent experiments. Results were shown as means ± SEM. Student’s t-test was used to evaluate the statistical significance. Statistical analyses were performed using nSolver and GraphPad Prism (version 8.4.3) analysis software. Overall survival and relapse-free survival (RFS) were performed using the Kaplan–Meier survival analysis and a *p*-value < 0.05 was considered significant (log-rank test).

## 3. Results

### 3.1. Identification of Differentially Expressed Genes (DEGs) Deregulated by WPS in HNOE Cells

**Figure 1:**
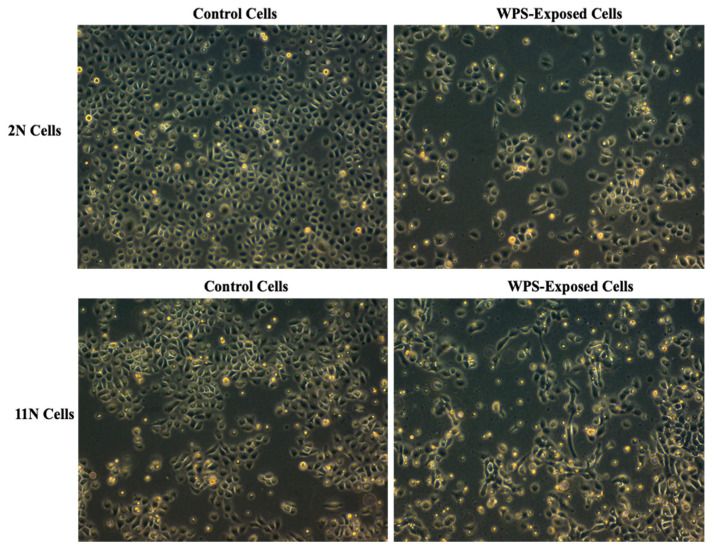
Water-pipe smoking (WPS) stimulates epithelial–mesenchymal transition (EMT) of human normal oral epithelial (HNOE) cell lines, 2N and 11N. We note that treatment for 2 days with 100 μg/mL of WPS solution induces morphological changes from epithelial (control) into a “fibroblast-like” (mesenchymal) phenotype, which is known as EMT.

In order to study the effect of WPS on human HN carcinogenesis, we examined the outcome of WPS on two human normal oral epithelial, 2N and 11N, which were established in our lab [28]. Our data revealed that treatment of 2N and 11N cell line with 100 μg/mL of WPS solution for 2 days slightly deregulates cell proliferation and cell cycle progression of both cell lines in comparison with untreated cells (data not shown). On the other hand, we found that WPS exposure induces EMT, where both cell lines display a more mesenchymal phenotype in comparison with their matched unexposed controls (Figure 1). The cells become more elongated in appearance and show a decrease in cell–cell contact compared with untreated ones (Figure 1).

Similar to our study, we previously examined the expression of E-cadherin and focal adhesion kinase (FAK) proteins in cancer cells exposed to WPS [27]. Our data showed loss of E-cadherin and enhanced expression of FAK proteins in WPS-exposed cells in comparison to unexposed ones; thus, indicating WPS promotes EMT progression and enhances cell migration as well as invasion abilities [27]. Furthermore, analysis of the underlying mechanisms revealed that the expression of phosphorylated Erk1/2 was upregulated in WPS-exposed cells, thus implying that WPS promotes EMT via Erk1/2 pathways [27,29,30].

Next, gene expression was applied on both cell lines, using the NanoString PanCancer Pathway Panel consisting of probes for 770 genes implicated in carcinogenic pathways; our data showed that out of these genes, 23 were found to be differentially expressed in WPS-exposed versus unexposed 2N and 11N cells: *CCL5, C1R, MMP9, IL-1B, CCL4, MASP2, OXER1, TLR3, STAT1, PPP1R12B, MX1, MX2, CCL21, IL-3, TLR9, HSH2D, CCR4, IFNγ, LT-β, IFIT1, TGF-β2, ALOX5*, and *LIMK1* (*p* < 0.05).

**Figure 2:**
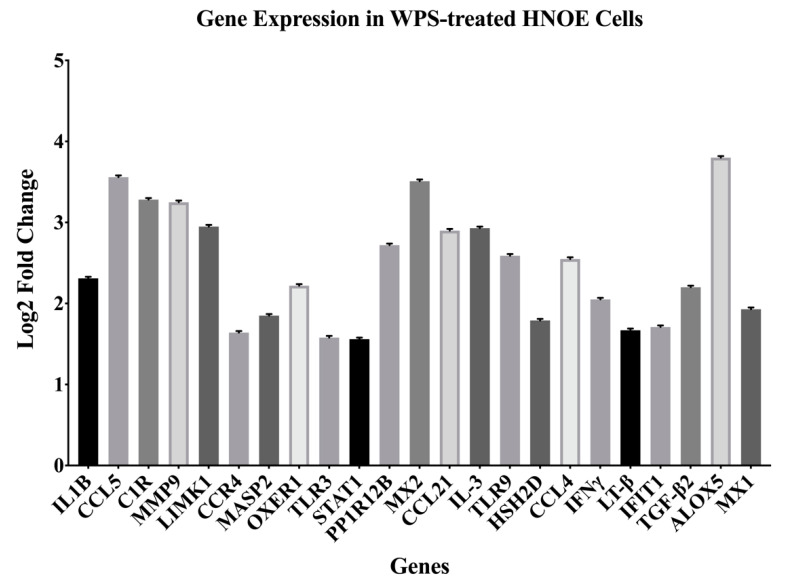
Differentially expressed genes (DEGs) discovered using the NanoString PanCancer Pathway Panel. Cut-offs used were 1.5-fold change or high between the different groups and adjusted for *p*-value < 0.05.

Following the identification of candidate genes, we validated our panel using qRT-PCR analysis. The set of genes differentially expressed corresponded with the NanoString analysis with twenty-three (*IL-IB, CCL5, C1R, MMP9, LIMK1, CCR4, MASP2, OXER1, TLR3, STAT1, PPP1R12B, MX2, CCL21, IL-3, TLR9, HSH2D, CCL4, IFNγ, LT-β, IFIT1, TGF-β2*, *ALOX5* and *MX1*) upregulated genes by a factor ranging from 1.58 to 3.8 folds (*p <* 0.05) (Figure 2).

Based on the molecular pathways of carcinogenesis, these 23 deregulated genes are directly involved in the modulation of cell cycle, proliferation, migration, invasion, apoptosis, angiogenesis, signal transduction, and inflammatory response (Table 2).

### 3.2. Deregulated Genes Are Upregulated in HN Cancer Samples Compared with Normal Tissue

Using the Oncomine database, we herein initially evaluated the mRNA expression levels of the DEGs discovered in normal tissue versus head and neck tumor samples.

Using the Toruner dataset (20 patient samples), we found that the expression of *IFIT1*, (*p =* 2.76 × 10^−8^) and *ALOX5* (*p* = 0.0019) genes were high in oral squamous cell carcinoma (OSCC) compared to normal squamous cells (Figure S1A). The Ginos dataset (54 patient samples) showed that *IL-1B*, (*p* = 1.77 × 10^−8^), *STAT1* (*p =* 9.25 × 10^−11^), *MX2* (*p* = 0.22 × 10^−8^), *LT-β* (*p* = 2.43 × 10^−6^), *C1R* (*p* = 0.005), *CCL5* (*p* = 2.28 × 10^−9^), *MMP9* (*p* = 7.07 × 10^−26^), *ALOX5* (*p* = 0.050) and *CCL4* (*p* = 3.20 × 10^−15^) genes were upregulated in HNSCC compared with the normal buccal mucosa (Figure S1B). Moreover, Cromer dataset (38 patient samples) reveled that *IL-1B* (*p* = 0.002), *STAT1* (*p* = 0.001) and *IFNγ* (*p* = 3.46 × 10^−4^) genes were high in HNSCC compared with normal uvula tissue (Figure S1C). The dataset (38 patient samples) exhibited *IL-1B* (*p* = 2.67 × 10^−6^), *TGF-β2* (*p* = 2.23 × 10^−4^), and *MMP9* (*p* = 0.01) genes to be upregulated in tongue squamous cell carcinoma (SCC) compared with normal tongue tissue (Figure S1D). In addition, the Peng dataset (79 patient samples) found *IL-1B* (*p = 4.52 × 10^−10^*), *IFIT1* (*p* = 1.27 × 10^−17^), *TGF-β2* (*p* = 2.23 × 10^−4^), *STAT1* (*p* = 7.18 × 10^−20^), *MX2* (*p* = 5.11 × 10^−11^), *OXER1* (122 patient samples, *p* = 0.045), *LT-β* (*p* = 0.004), *CCL21* (122 patients, *p* = 0.005), *C1R* (*p* = 0.005), *CCR4* (*p* = 2.50 × 10^−5^), *HSH2D* (*p* = 2.47 × 10^−7^), *MASP2* (*p* = 5.16 × 10^−5^), *PPP1R12B* (*p* = 4.10 × 10^−4^), *CCL5* (122 patients, *p* = 3.33 × 10^−12^), *MX1* (*p* = 1.70 × 10^−9^), and *IFNγ* (*p* = 6.82 × 10^−13^) genes to be over expressed in OSCC compared with normal oral cavity (Figure S1E). Sengupta dataset (41 patient samples) evaluated that expression of *IFIT1* (*p* = 5.38 × 10^−5^), *CCR4* (*p* = 0.046), and *CCL4* (*p* = 2.79 × 10^−8^) genes were increased in nasopharyngeal carcinoma compared with normal nasopharynx (Figure S1F). Estilo dataset (58 patients) showed *STAT1* (*p* = 1.07 × 10^−12^), *MX2* (*p* = 4.53 × 10^−5^), and *MX1* (*p* = 1.31 × 10^−10^) genes to be highly expressed in tongue SCC compared with normal tongue tissue (Figure S1G). While *IL-3* expression in Kuriakose dataset (20 patient samples) was predominant in lip and OSCC (20 patients, *p* = 0.009) (Figure S1H). Additionally, *OXER1* (*p* = 1.38 × 10^−9^) expression using TCGA dataset is highly upregulated in HNSCC (364 patient samples) (Figure S1I).

### 3.3. Deregulated Genes Are Upregulated in Smoking HN Cancer Patients Compared to Non-Smoker Patients

Next, and to further investigate the association between our discovered DEGs and smoking as a risk factor of cancer, we investigated the DNA copy numbers of the 22 upregulated DEGs in HN cancer samples in smoker versus non-smoker HN cancer patients. For this analysis, we used TCGA HN dataset (270 patients) of the Oncomine database. Interestingly, our results confirmed that, of the 22 genes, 16 were upregulated in smoking HN cancer patients compared to those who had never smoked. These genes include *CCL5, C1R, MMP9, IL-1B, CCL4, OXER1, TLR3, STAT1, PPP1R12B, MX1, MX2, HSH2D, IFNγ, LT-β, IFIT1*, and *TGF-β2* (*p ≤ 0.05*) (Figure S2).

### 3.4. Deregulated Genes by WPS Have a Direct Impact on HN Cancer Patient’s Prognosis

Subsequently, we explored whether the DEGs induced by WPS in oral epithelial cells could have an impact on the prognosis of HN cancer patients. To asses this point, we analyzed the association between the DEGs mRNA expression and patient’s outcome, relapse-free survival (RFS), or overall survival (OS), using a large HNSCC cohort (n = 500 patients) from the Kaplan–Meier plotter database.

**Figure 3:**
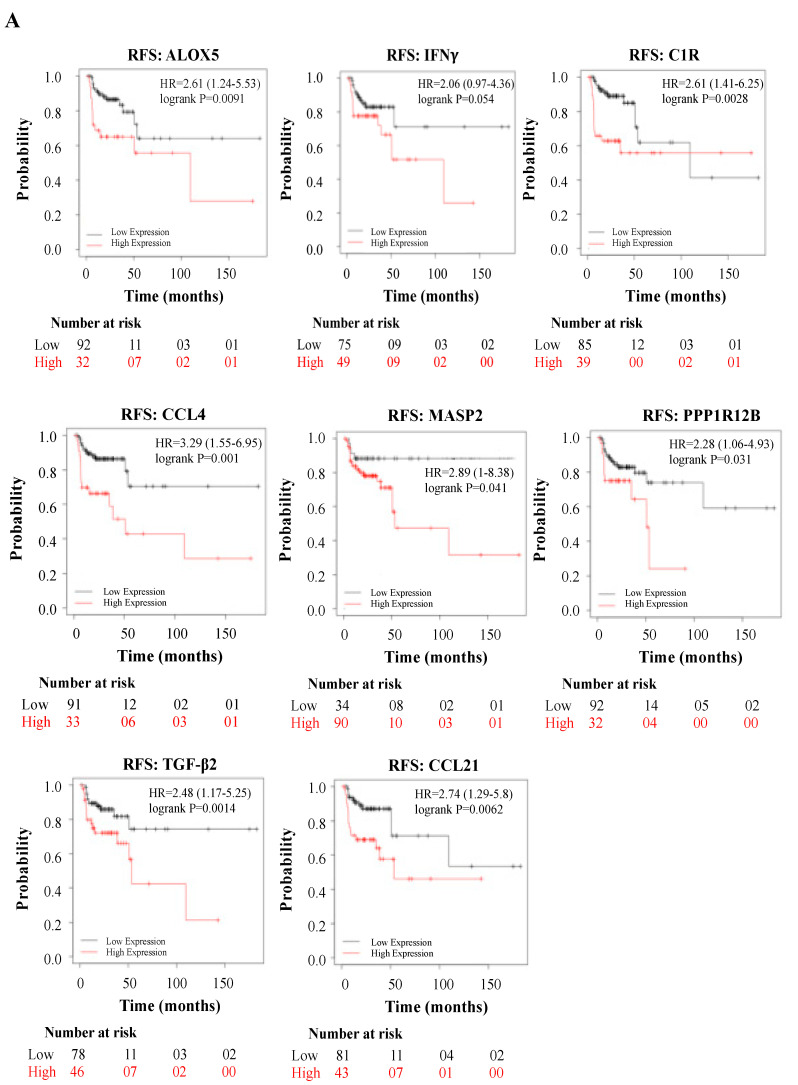

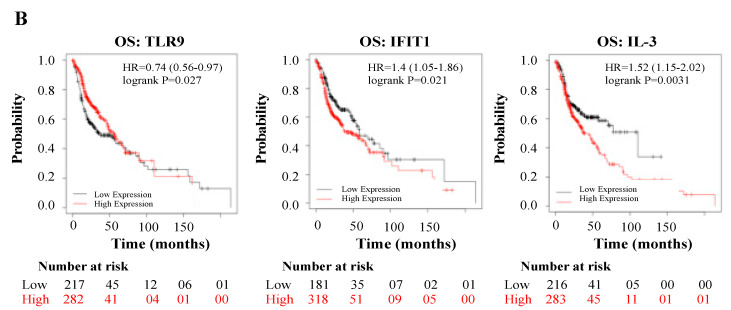
(**A**,**B**): Relapse-free survival (RFS) in HN cancer patients using the Kaplan–Meier plotter database, expressed by relapse-free survival (RFS) and overall survival (OS).

Interestingly, while high expression of *ALOX5* (*p* = 0.0091), *IFNγ* (*p* = 0.054), *C1R* (*p* = 0.0028), *CCL4* (*p* = *0.001*), *MASP2* (*p* = 0.041), *PPP1R12B* (*p* = 0.031), *TGF-β2* (*p* = 0.014), and *CCL21* (*p* = 0.0062) correlates positively with poor RFS (Figure 3A), expression of *TLR9* (*p* = 0.027), *IFIT1* (*p* = 0.021) and *IL-3* (*p* = 0.0031) correlates positively with poor OS (Figure 3B).

### 3.5. WPS Deregulated Genes Are Mainly Involved in Immune Response and Cytokine/Chemokine Mediated Pathways

**Figure 4:**
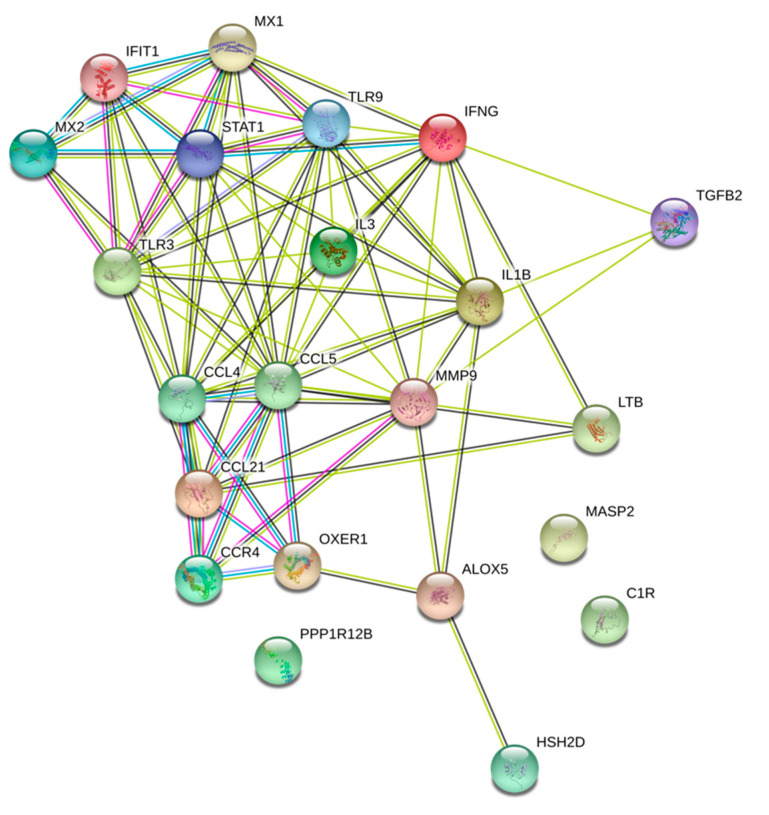
Protein interaction analysis of the WPS upregulated and differentially expressed genes using the Search Tool for the Retrieval of Interacting Genes (STRING v9.1). Enriched biological process and molecular functions of those proteins are included.

We investigated major gene interactions between top DEGs and possible pathway enrichment. Interestingly, our results showed a strong interaction with major biological processes including immune response, cytokine-mediated signaling pathway, and cellular response to cytokine stimulus. Moreover, the molecular functions shared between the top DEGs were also found to be related to cytokine and Cysteine-Cysteine Chemokine Receptor (CCR) chemokine binding receptors (Figure 4), (Table 3).

## 4. Discussion

This investigation, to the best of our knowledge, is the first cancer genes profiling study on the effects of WPS exposure on human normal oral epithelial cells. Previously, our group has revealed that WPS can play an important role in the initiation and progression of human oral cancer, which represents the majority of HN cancer cases [24]. In this study, we found that WPS can induce EMT, which is the hallmark of cancer progression in human normal oral epithelial cells by loss of E-cadherin and upregulation of FAK protein as well as Erk1/2 pathways as previously demonstrated in our study [27,29,30]. More importantly, we used a NanoString nCounter PanCancer Pathways panel of 770 gene transcripts distributed in 13 biological pathways to determine gene targets of WPS exposure in human normal oral epithelial (HNOE) cells. Thus, we identified significant changes in the expression of 23 genes, with one gene being down-regulated and twenty-two being upregulated. In our investigation, we confirmed, both by qRT-PCR as well as the Oncomine TM database, the deregulation of the newly identified genes as targets for WPS exposure in oral cells. We also analyzed the prognostic effect of the WPS-induced deregulated genes on HNSCC survival and prognosis using Pan-cancer RNA-seq dataset of the Kaplan–Meier plotter database. More significantly, our study points out that these genes are discovered for the first time as targets of WPS exposure in human normal oral epithelial cells. The uncovered genes encode for proteins that are known for regulating cell cycle, proliferation, migration, invasion, apoptosis, angiogenesis, signal transduction, and inflammatory response. Therefore, the newly identified genes can plausibly play a role in the neoplastic transformation of normal oral epithelial cells and consequently HN cancer initiation in general.

Of the nine differentially expressed genes, four (*CCL5, CCL21, CCL4*, and *CCR4*) belong to the family of chemokines. Elevated *CCL5* levels are significantly associated with oral cancer progression [31], relapse, and/or metastasis [32,33], as well as drug resistance [34], indicating its fundamental role in oral carcinogenesis [35]. Our results are concordant, suggesting *CCL5* association with oral cancer progression upon WPS intake. Moreover, *CCL5* is capable of upregulating the release of MMP-9 [36], a matrix-metalloproteinase that was also identified in our study. *CCL5* enhances oral cancer cell migration through the increase in *MMP-9* production [31]. Earlier studies have shown that overexpression of *MMP9* is observed in oral cancer [37] and is associated with a poor disease-free survival (DFS) [38]. Similar data were observed in this study. Interestingly, *ALOX5* acts as a mediator of invasion via *MMP-9* induction [39]. *ALOX5* expression is known to be involved in carcinogenesis [39] and is also involved in chronic obstructive pulmonary disease (COPD) [40]. Additionally, *ALOX5* genotype was found to be linked with asthma and poorer lung function [41], while its expression in mice models showed increase in inflammation, oxidative stress as well as emphysema caused by cigarette smoke [42], indicating its possible involvement in oral cancer upon exposure to smoke from WPS. Although a previous study has shown an association between *ALOX5* with poor asthma controls [41], there are no studies indicating association of *ALOX5* with RFS; we herein show for the first time that *ALOX5* is associated with shorter RFS.

Similar to *CCL5*, *CCL4* has analogous role in cancer progression; *CCL4* enhances susceptibility to oral cancer [43]. It has been shown that *CCL4* stimulates *VEGF-C* expression by activating the JAK2/STAT3 signaling pathway, which is frequently linked with oral cancer cell proliferation, invasion, and angiogenesis [44]. A recent investigation showed that smoking along with *CCL4* gene polymorphisms can increase risk of oral cancer [43]. Likewise, we found that WPS smoking can augment *CCL4* expression resulting in enhanced inflammatory response, thus promoting tumor development and progression. Similar to our data, *CCL4* expression is linked with poor prognosis in cancer [45]. On the other hand, cigarette smoking has been shown to increase blood and bronchoalveolar lavage fluid levels of the CCR7 ligands *CCL19* and *CCL21* [46], thereby contributing to migration of lung cancer cells [47]. Additionally, previous research has considered *CCL21* in oral cancer as a candidate marker for unfavorable outcome [48]. In this study, we confirm that *CCL21* is a target of WPS exposure in oral epithelial cells, implying its possible role in cell transformation and therefore HN carcinogenesis. Concordant to our data, recently it has been pointed out that CCL21/CCR7 is linked with cancer recurrence, smoking, and poor prognosis in HN cancer [49,50]. In this regard, chemokine receptors are G protein-coupled receptors and are involved in the onset and progression of several solid tumors [51,52,53]. Concordant to our data, several investigations reported *CCR4* to play a role in lymph node metastasis of HNSCC as well as its progression and recurrence [50,54]. Moreover, we also identified upregulated expression of G protein coupled *OXER1* in HNSCC. *OXER1* has been reported to be upregulated in both prostate cancer cells as well as tumor tissues [55]. However, although, no direct role of *OXER1* has been studied in HNSCC, upregulation of *OXER1* in human papillomavirus (HPV)-positive tumors has been previously reported [56]. Since HPV is found to play a role in the onset and progression of HN as well as oral cancers [28,57,58], we suggest a link between *OXER1* expression and HN as well as oral cancers.

Moreover, in this study, of the 23 genes, 4 (*TLR3, TLR9, C1R* and *MASP-2*) of them are involved in innate immune system. We reveal that *TLR3* and *TLR9* are upregulated in WPS-exposed oral epithelial cells. It has been demonstrated that HN cancer cell lines as well as OSCC tissue samples express TLR3 thereby enhancing the expression levels NF-κB and its regulated oncogene, *c-myc*, thus inciting cellular proliferation and migration, which is significantly associated with poorly differentiated tumor cells and perineural invasion [59,60,61,62]. Similar to data obtained in this study, upregulated expression of *TLR9* in HNSCC as well OSCC was found to promote tumor cell invasion, proliferation as well as migration by enhancing MMP-2 expression [63,64,65,66]. Although *C1R* is known to regulate the complement pathway of the innate immune system, in this study, we found upregulated *CIR* expression in HNSCC. This is in concordance with a previous study that correlated the expression of *C1R* in cutaneous SCC (cSCC) with tumor progression, cell proliferation, and migration [67,68]. Since cSCC lesions frequently develop in the HN region [69], our data correlate with this finding. Although, the prognostic relevance of *C1R* has not been studied in cancer; however, it is associated with tumor progression and migration [67,68], hence our data suggest a significant correlation between C1R and shorter RFS. Furthermore, our data implicate the other member of the complement pathway, *MASP-2* gene, which is upregulated in oral epithelial cells exposed to WPS. *MASP-2* produced in hepatocytes is involved in innate response, and its promoter is regulated by cytokines (interleukins and TGF-β) or transcription factor (STAT) [70]; our study found both cytokines and STAT to be expressed in WPS exposed HNOE cells. Previous studies have found an association between *MASP-2* expression and cancer [71,72,73]; *MASP-2* expression significantly correlates with late clinical stage and nodal metastasis, thus indicating its role in cancer progression and aggressive tumor behavior in esophageal SCC [71]. Moreover, *MASP-2* is significantly associated with recurrence and poor survival of colorectal as well as ovarian cancers [72,73], thus suggesting a link with poor RFS, similar to data found in this study.

The pro-inflammatory cytokine, *IL-1B*, is elevated in HNSCC including oral cancer [74,75]. Furthermore, similar to our data, an earlier study demonstrated the upregulation of *IL-1B* in tobacco and betel quid-mediated OSCC; *IL-1B* promotes proliferation of dysplasia of oral cells, thus triggering oncogenic cytokines as promoters of tumor aggressiveness [76]. On the other hand, cytokine *IL-3*, is a selective growth factor that stimulates tumor angiogenesis [77]. However, although the role of *IL-3* in COPD as well as cigarette smoking is not well defined, *IL-3* levels were previously detected in SCC [78]. We herein show presence of *IL-3* in oral epithelial cells under the effect of WPS, thus indicating its role in oral cancer progression. Although data have shown strong correlation between *IL-3* and poor survival in acute myeloid leukemia [79], research on the role of *IL-3* in OSCC is scarce. Interestingly, a recent study by Almeida et al. (2019) showed an association between *IL-4, IL-6, IL-8, IL-10, IL-12*, and *IL-13* and poor survival in OSCC [80]. In this study, we found *IL-3* to significantly correlate with poor overall survival, which requires further investigation. On the other hand, transforming growth factor (TGF), a cytokine, is involved in promoting cellular invasion as well as angiogenesis in OSCC cells [81,82]. Our data revealed the presence of TGF-β2 in WPS-exposed oral epithelial cells as well as in HNSCC; previous studies have indicated the presence of TGF-β2 in cancer associated fibroblasts from OSCC [83] as well as in SCC cell lines [84]. Similar to our data, elevated *TGF* expression was significantly associated with shorter OS, RFS, and DFS in patients with OSCC [85]. The other type of cytokine identified in this study is *IFNγ*. In oral epithelial cells, a loss of *IFNγ* expression may be caused by the *IFN-γ* promoter methylation as a plausible underlying mechanism for oral cancer progression [86]. Furthermore, and in concordance with our data, low *IFNγ* levels are associated with poor prognosis in HNSCC including oral [87,88]. The other cytokine, *LT-β*, was also identified in this study; *LT-β* has been shown to correlate with human oral cancer [89], and additionally, it activates the NIK-IKKa-RELB/NF-κB2 pathway to stimulate HNSCC cell migration [90,91]. While *STAT1* has a dual role in HNSCC [92,93,94], we report upregulation of *STAT1* in OSCC as well as HNSCC, as reported previously [92], suggesting an oncogenic role of *STAT1* in the pathogenesis of HNSCC.

On the other hand, we report the upregulation of an interferon-stimulated gene, *IFIT-1*, in WPS-exposed HNOE cells. In this context, a previous study showed that over-expression of *IFIT1* in OSCC cells promote tumor growth and metastasis by activating *EGFR* signaling [95]. Research in OSCC has shown a distinct correlation between elevated *IFIT1* expression with T-stage, lymph node metastasis, lymphovascular, perineural invasion, as well as poor overall survival in OSCC patients [95]. Moreover, increased *IFIT1* expression in OSCC cells enhance resistance to several therapeutic agents (5-FU, carboplatin, cisplatin, ganetespib, and oxaliplatin) [96,97], thus indicating its role in poor RFS, which is similar to data obtained in our study. We identified the interferon-related gene, *MXI* along with its paralogue *MX2. MX1* has a contradictory role in cancer; in one study, *MX1* is upregulated in OSCC [98], but nevertheless, it is hyper methylated in HN cancer [99]. However, in our study, we found that exposure to WPS smoke in oral epithelial cells induced expression of *MX1*, indicating its possible oncogenic role in oral cancer.

Interestingly, we discovered two genes (*HSH2D* and *PPP1R12B*) in our cohort that were not previously reported, as possible players in OSCC or HNSCC. Traditionally, *HSH2D* has a role in T-cell activation and is a downstream target of CD28 costimulatory signaling pathway [100]. A previous study has reported loss of CD28 on T-cell in HNSCC [101]; thus, we postulate a role of *HSH2D* activation in HNSCC. Moreover, *PPP1R12B*, also known as *MYPT2*, is a subunit of MYPT [102]. Although no direct role of *MYPT2* is implicated in cancer, *MYPT* is found to be involved in cancer [103,104], indicating a plausible role for *MYPT2* in HNSCC. We found *PPP1R12B* to be associated with poor RFS. However, studies have shown *MYPT* to correlate significantly with drug resistance and poor prognosis in human carcinomas [103,104], thus postulating a plausible role of *PPP1R12B* in drug resistance and poor prognosis in OSCC or HNSCC.

An earlier study by our group show clearly that WPS stimulate cell invasion of human breast cancer cells [27]. While several investigations have shown a significant correlation between smoking and the onset/progression of oral cancer [27,105,106,107,108]. In addition, it has been revealed that cigarette smoking can enhance EMT of several human carcinoma cells [29,109,110,111,112]. Thus, it is apparent that smoking is an important etiological factor in the onset of numerous human cancers inducing lung, HN (especially oral) as well as breast [27,105,113,114,115]. Nevertheless, based on the number and level of toxicants and the duration of smoking session, it can be assumed that WPS is more harmful with regards to the development and progression of human cancers as well as cancer-related deaths in comparison with cigarette smoking.

## 5. Conclusions

We reveal for the first time, that WPS can induce EMT in human normal oral epithelial cells, which is accompanied by the deregulation of a set of genes related to oncogenesis. Thus, WPS can promote HN cancer initiation and/or progression mainly due to its effect on key regulatory genes of carcinogenesis that have a direct impact on HN cancer patients’ outcome. Nevertheless, further studies are needed to elucidate the expression of different proteins involved in EMT as well as to understand the full mechanism by which WPS can induce HN carcinogenesis.

## Figures and Tables

**Table 1:** List of primers sequences used for reverse transcriptase real-time PCR.

Gene	Forward (5′-3′)	Reverse (5′-3′)
***CCL5***	GGTGCCAGCAAGATAACCCT	GCTTGCCTGACTTCCTCCTT
***MX1***	AGGTTCCAGTAGGGCATGTG	TTGGAAAGAAGGTGCTTGCT
***CCL21***	CTGGACAAGACACCATCCCC	TGTACTGGGGAGCCGTATCA
***IFNγ***	CTCATGTAAGCCCCCAGAAA	GCCCAGTTCCTGCAGAGTAG
***ALOX5***	ACTTCGCCGACTTTGAGAAA	CAAGGGTGACCACAGTGATG
***MMP9***	GTCTTGTGGAGGCTTTGAGC	CAGGGATCTCCCCTCCTTAG
***CCL4***	GCTAAATCCAGTGGGTGGAA	GCTTGCTTCTTTTGGTTTGG
***IL-3***	GTAGAGACGGGGTTTCACCA	GGCACAGGCCTAGAAGTGAG
***TLR9***	CAGCAGCTCTGCAGTACGTC	AAGGCCAGGTAATTGTCACG
***IL-1B***	GGCTGCTGACTTTGAAGGAC	CATGGGAAGAAACTGGGAGA
***LIMK1***	TCTGCAAGTGTTCGCCATAG	AGGGAGGCTCTGAAGGAAAG
***C1R***	GTTTTGGCAGGTGGCTCTTG	AGGCACAGTGGTTTCCCAAA
***MASP2***	CCCTGGAGATTGATTCCTCA	AAACCCACTGGTCAGTTTCG
***OXER1***	GAAACCCACCTAGGCCTCTC	TTGGAAGGGACAAACTGGAG
***TLR3***	AGCCTTCAACGACTGATGCT	TTTCCAGAGCCGTGCTAAGT
***STAT1***	GCAGAGACATGCCTTTGTCA	GCCACTCAGCTATTGCTTCC
***PPP1R12B***	CCAAGTTGATTCAAGCAGCA	GTTCAAGTCCAGGGCAACAT
***MX2***	AGGTTCCAGTAGGGCATGTG	TTGGAAAGAAGGTGCTTGCT
***HSH2D***	CCACGCATGTAGGGAAGTTT	AGGGTCAGGGCTGTGTTATG
***CCR4***	GTACTCCAACCTGGGCAAAA	CAGACTGGGTGACAGAGCAA
***LT-β***	AGGAGCCACTTCTCTGGTGA	AAAAGACCACAGGCACAACC
***IFIT1***	CTGTGGTAGGCTCTGCTTCC	CCACCACACCCAGCTAAGTT
***TGF-β2***	GGCAAATAGCCTGGTGTTGT	GCTGAGTTGGCATTCTGACA

**Table 2:** Genes based on their functional annotations.

Molecular and Cellular Functions	Genes Involved
Cellular Processes (Cell Cycle, Proliferation, Migration, Invasion, Apoptosis, and Angiogenesis)	*CCR4, IL-1B, IL-3, LIMK1, MMP9*
Signal Transduction	*CCL4, CCL21, HSH2D, IFNγ, IFIT1, MASP2, MX1, MX2, OXER1, PPP1R12B, STAT1, TGF-* *β* *2, TLR9*
Inflammatory Response	*ALOX5, C1R, CCL4, IFNγ, LT-β*

**Table 3:** Functional annotations of the differentially expressed genes.

**Molecular Function (GO)**			
**Go-Term**	**Description**	**Count in Gene Set**	**False Discovery Rate**
GO:0005126	Cytokine receptor binding	10 of 272	6.46 × 10^−11^
GO:0005125	Cytokine activity	8 of 216	6.87 × 10^−9^
GO:0048020	CCR chemokine receptor binding	4 of 41	5.61 × 10^−6^
GO:0031730	CCR5 chemokine receptor binding	3 of 7	5.61 × 10^−6^
GO:0005149	Interlukin-1 receptor binding	3 of 18	3.85 × 10^−5^
**Biological Process**			
**Go-Term**	**Description**	**Count in Gene Set**	**False Discovery Rate**
GO:0006955	Immune response	18 of 1560	1.54 × 10^−13^
GO:0002376	Immune system process	20 of 2370	1.54 × 10^−13^
GO:0019221	Cytokine-mediated signaling pathway	14 of 655	3.65 × 10^−13^
GO:0071345	Cellular response to cytokine stimulus	15 of 953	1.22 × 10^−12^
GO:0006952	Defense response	16 of 1234	1.22 × 10^−12^

## Supplementary Materials

The following are available online at https://www.mdpi.com/2305-6304/8/3/73/s1, Figure S1: mRNA expression levels of the differentially expressed genes (DEGs) in normal tissue compared with head and neck cancer, using (**A**) Toruner, (**B**) Ginos, (**C**) Cromer, (**D**) Ye, (**E**) Peng, (**F**) Sengupta, (**G**) Estilo, (**H**) Kuriakose, and (**I**) TCGA datasets. Figure S2: DNA copy number of the top WPS differentially expressed genes (DEGs) in smoker versus never smoker HN cancer patients using TCGA HN dataset (270 patients) using the Oncomine database.
