# Supplementary figures and images for: Water-Pipe Smoking Exposure Deregulates a Set of Genes Associated with Human Head and Neck Cancer Development and Prognosis
Source: Toxics. 2020 Sep 18;8(3):73. doi: 10.3390/toxics8030073 (PMC7560251; doi:10.3390/toxics8030073)

**A**

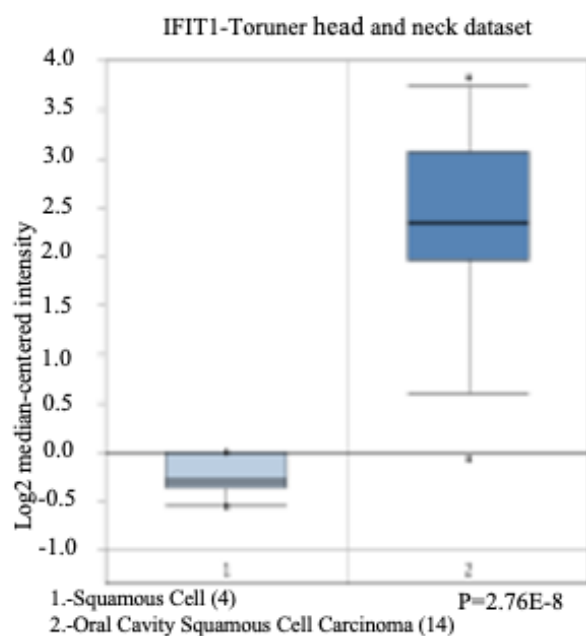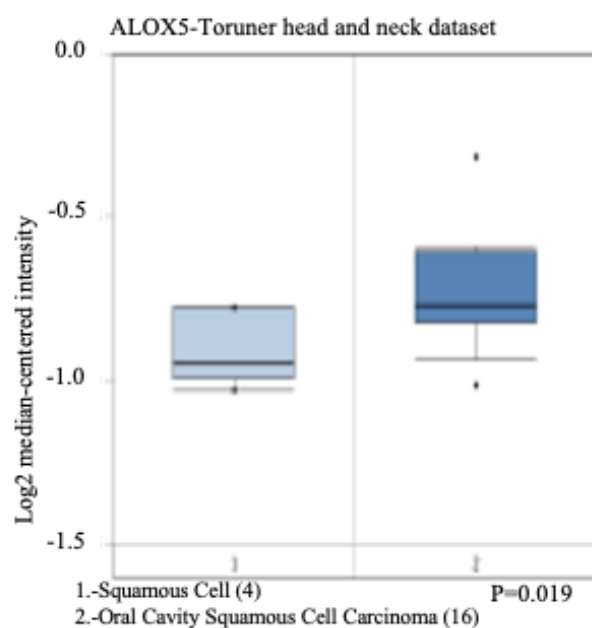

**B**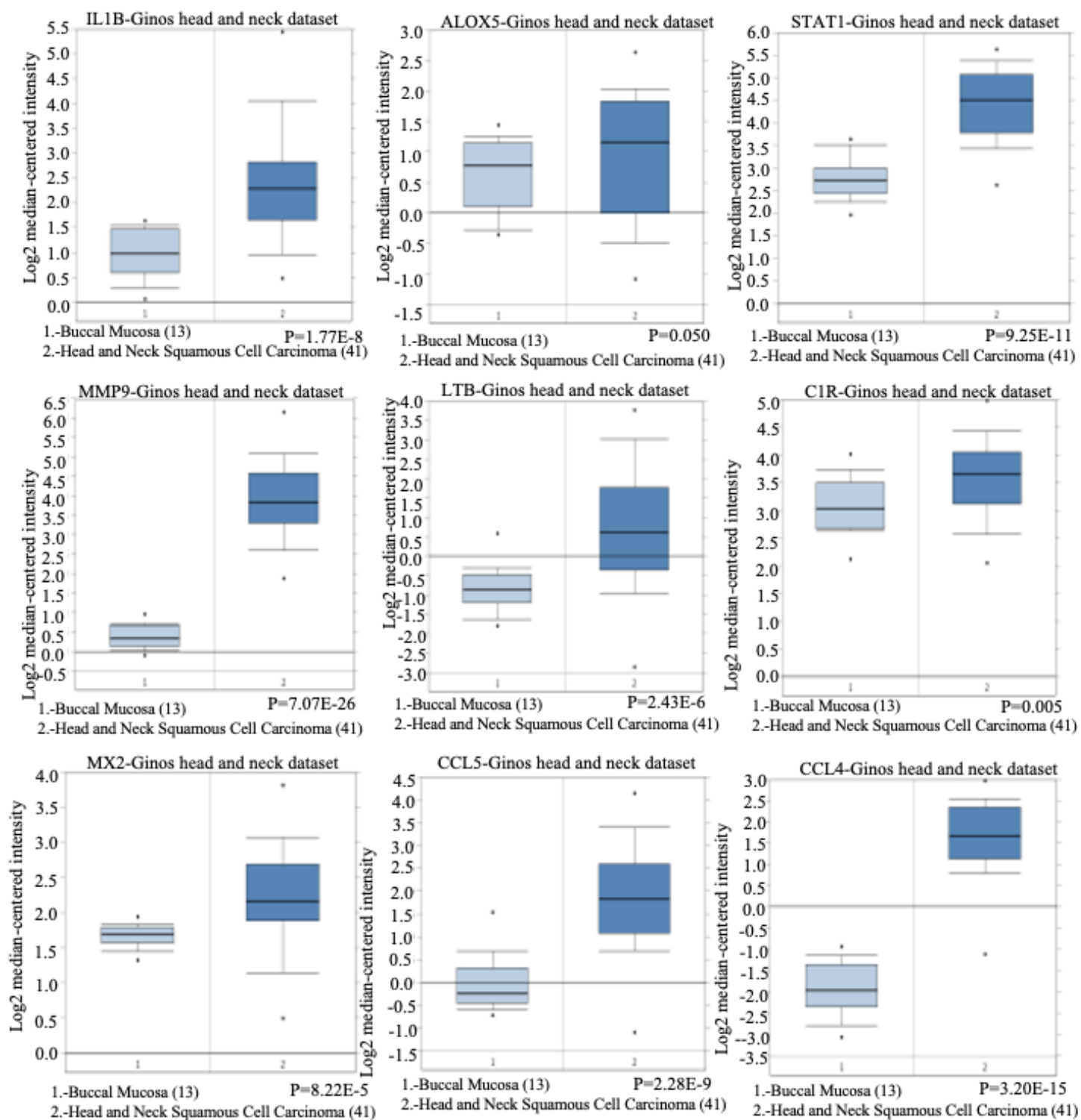

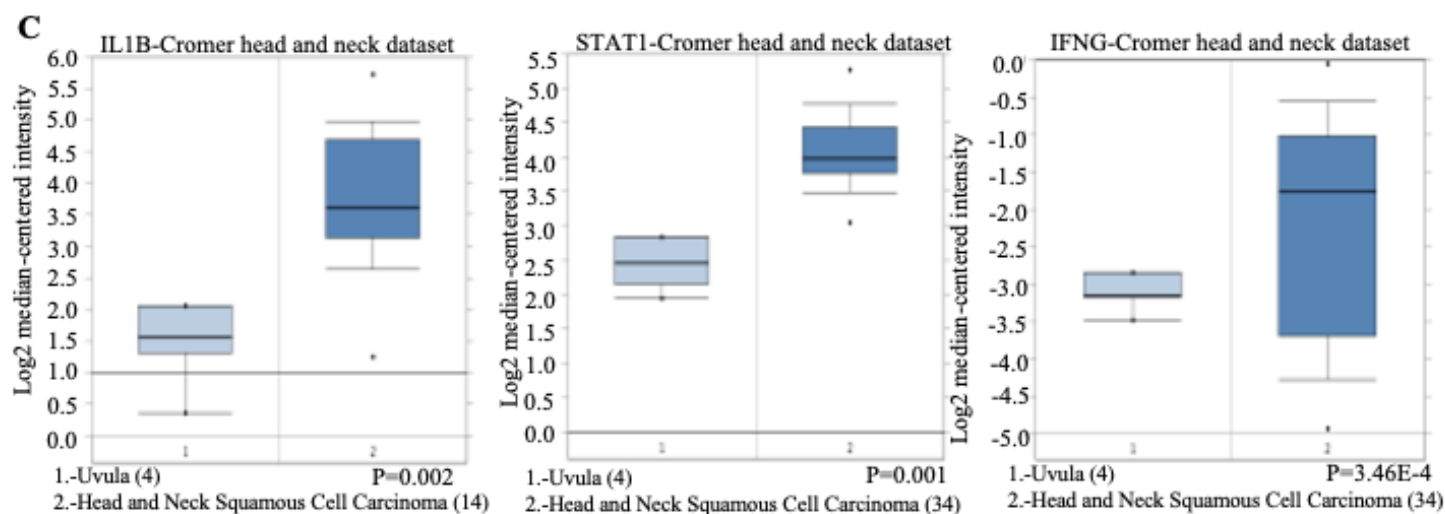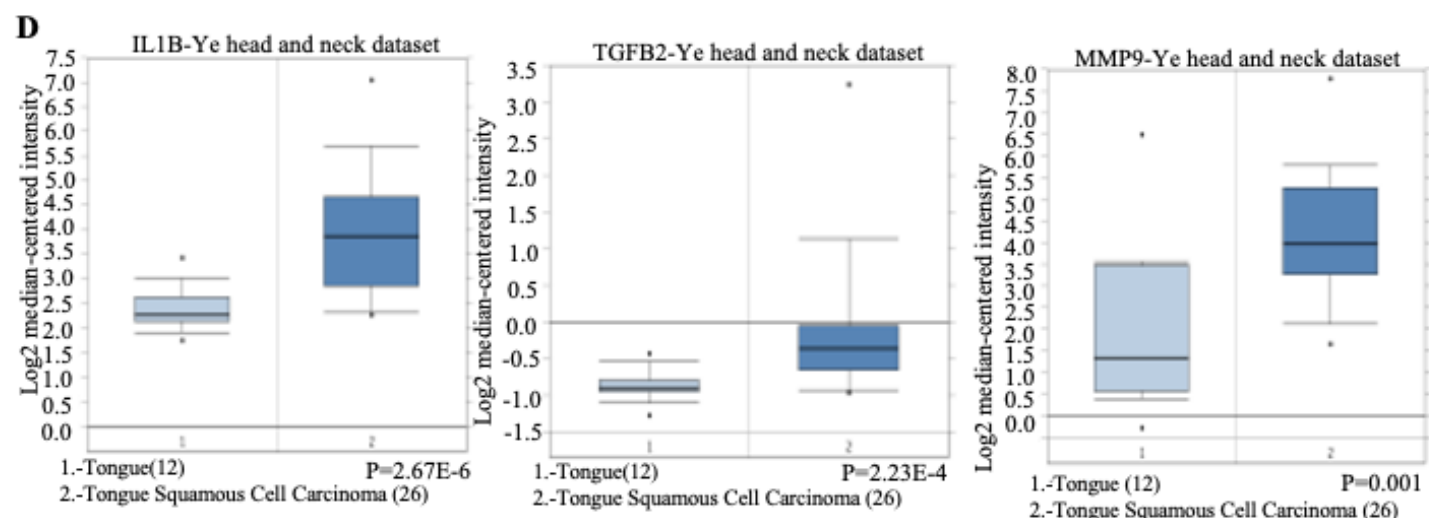

**E**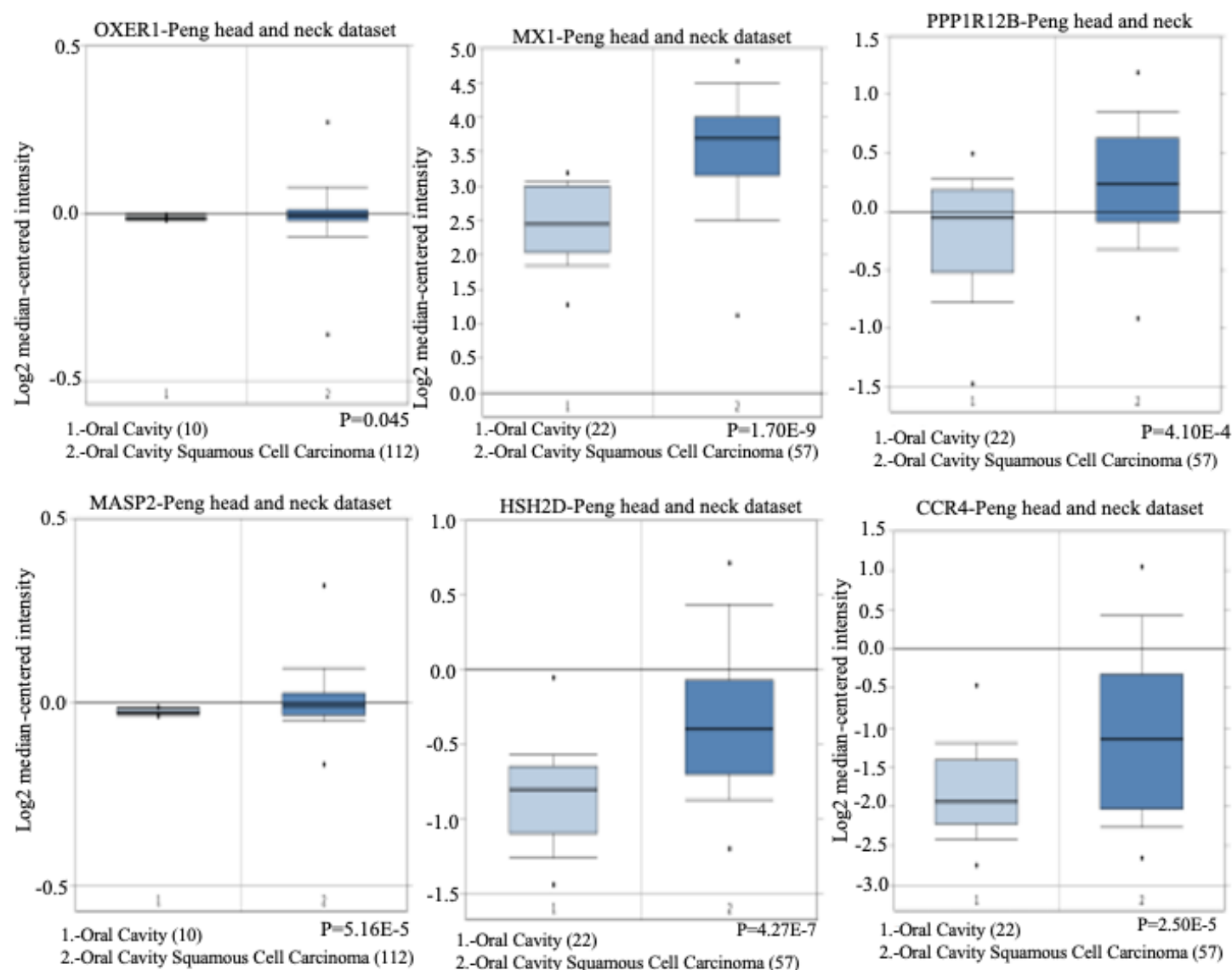**F**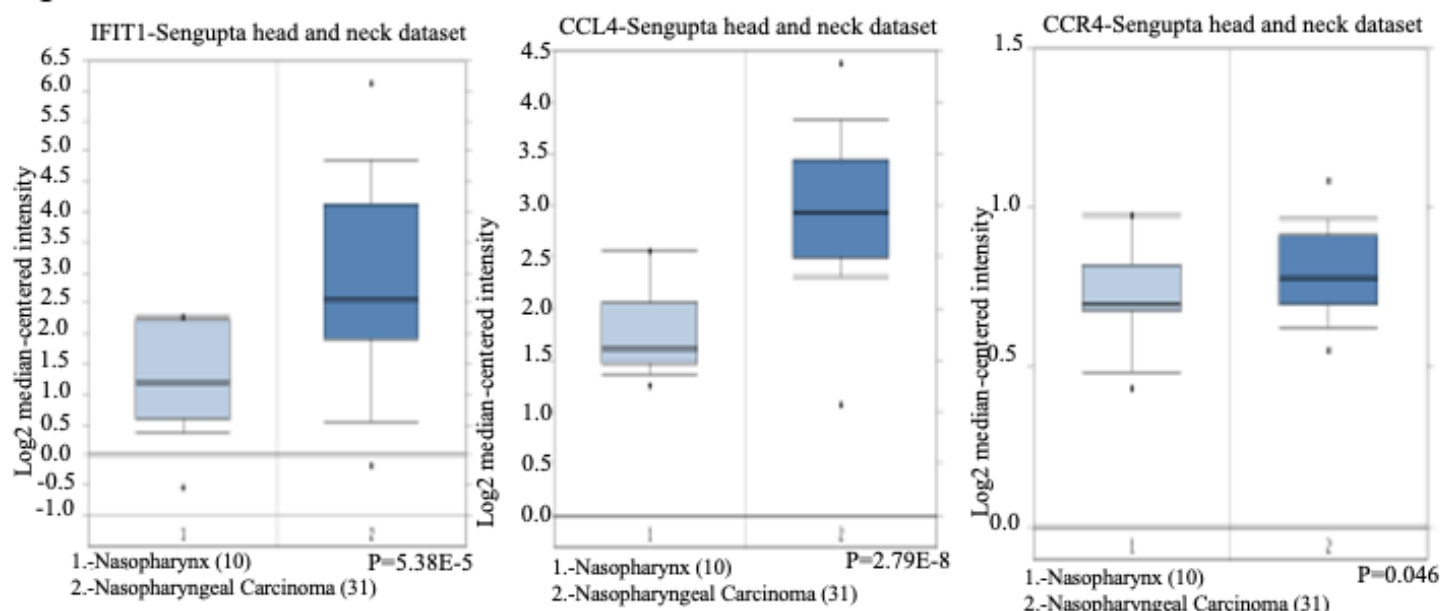

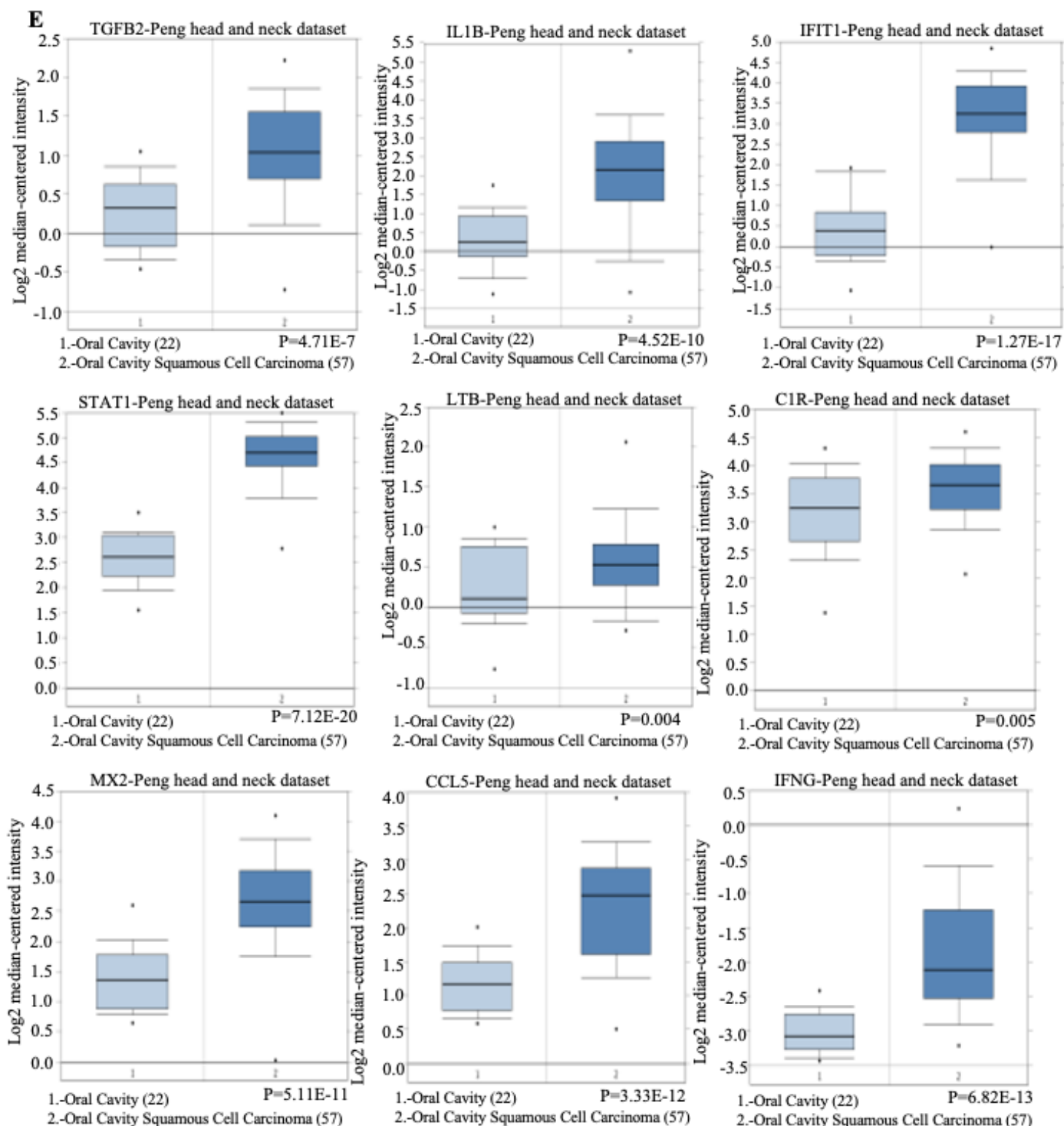

**G**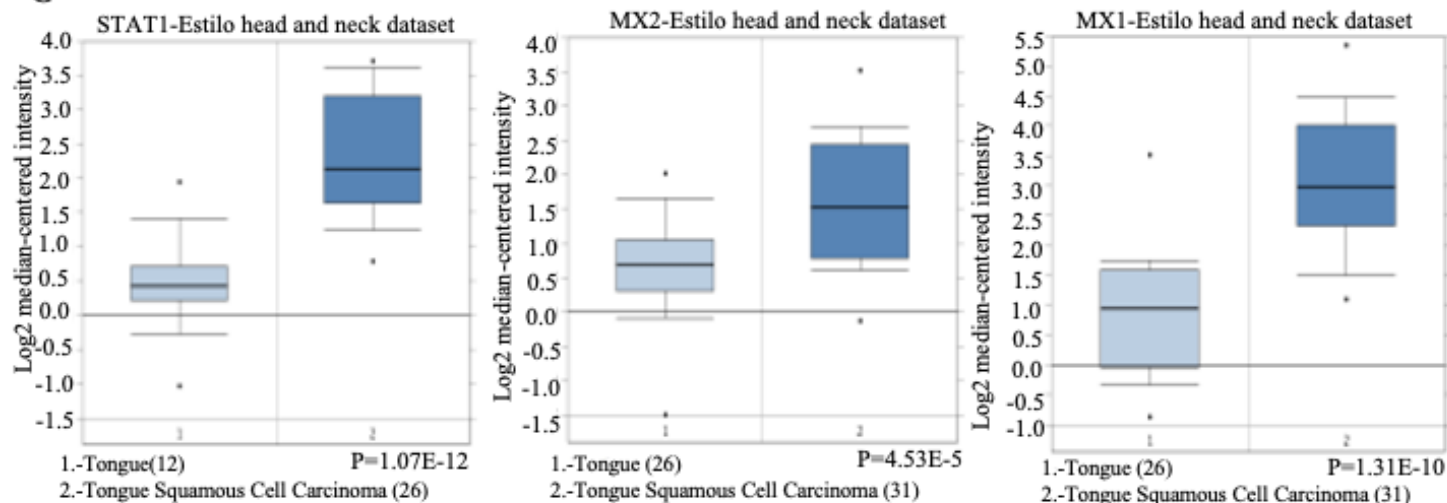**H**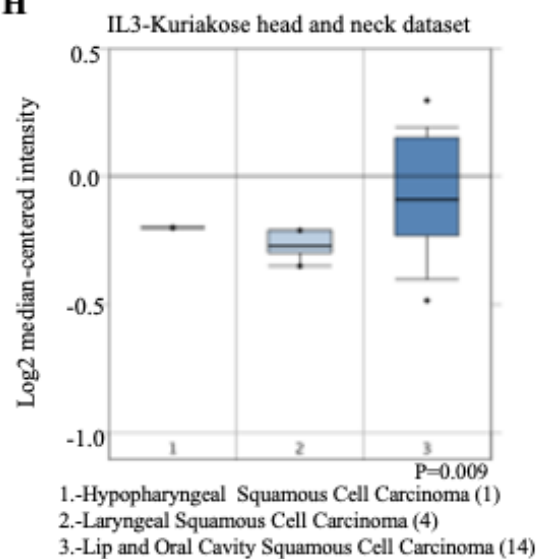**I**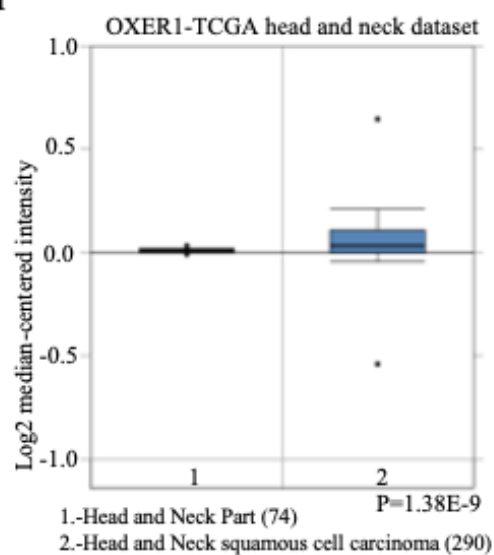

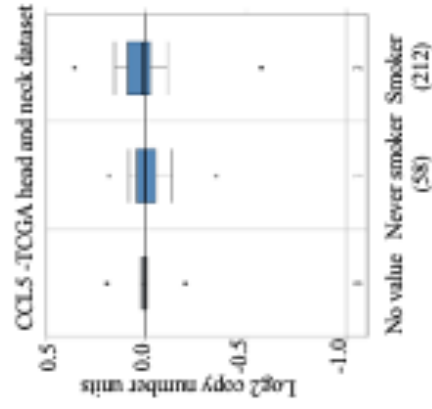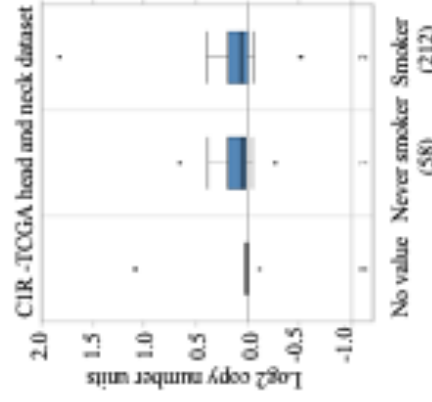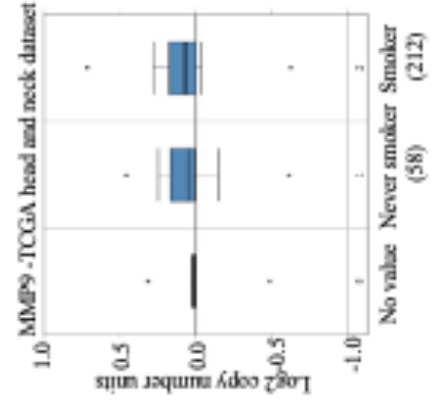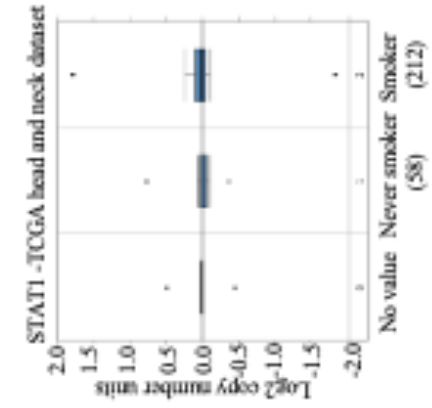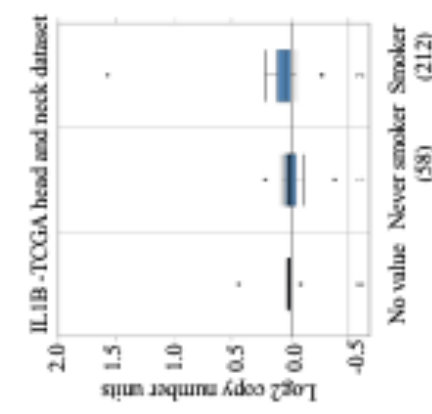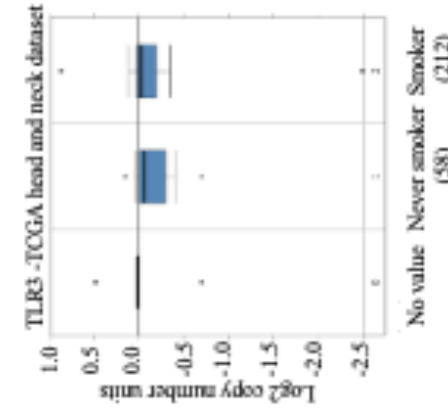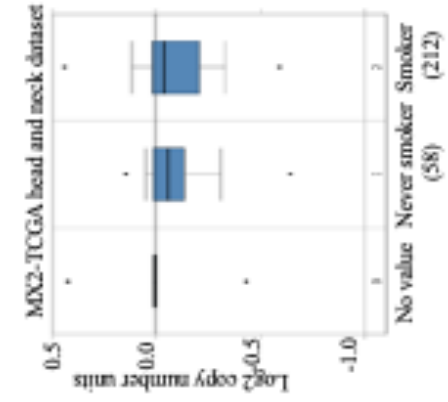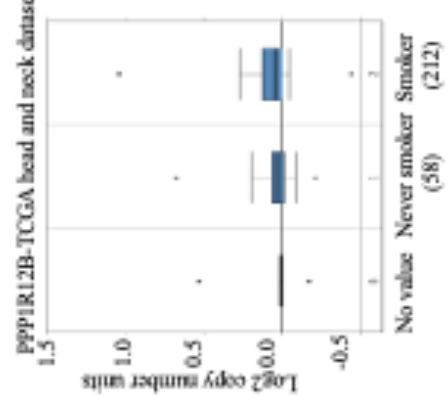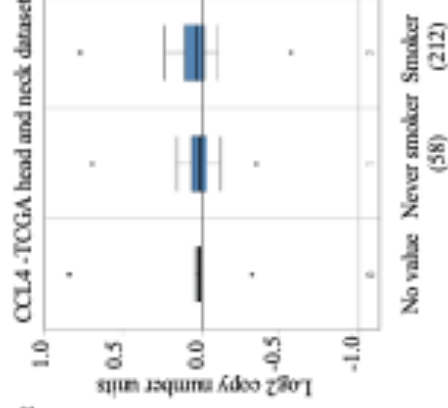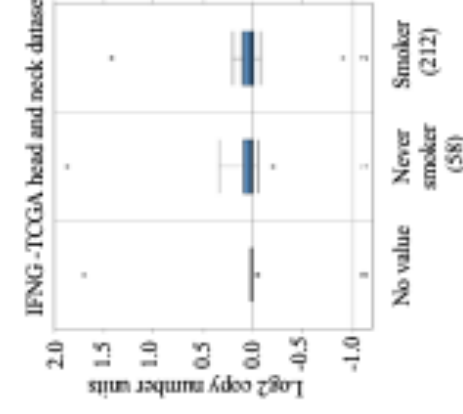

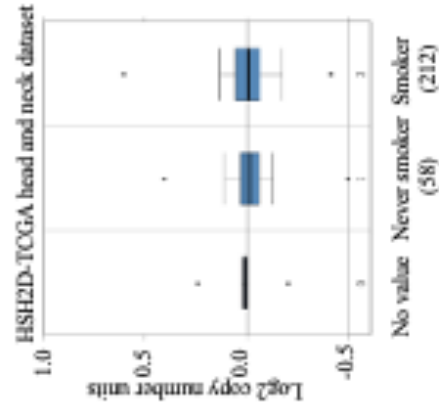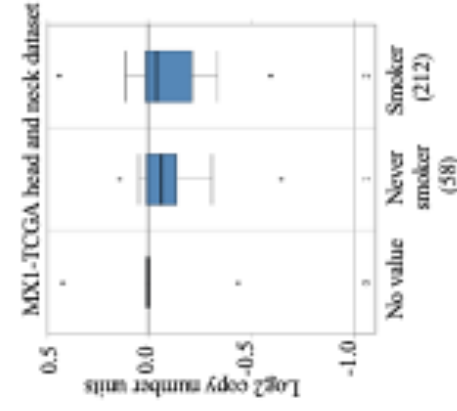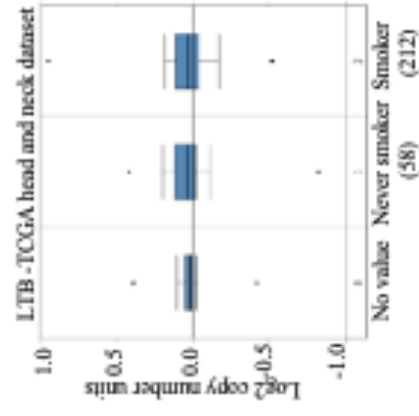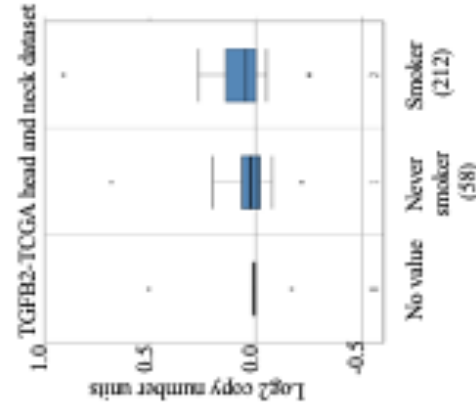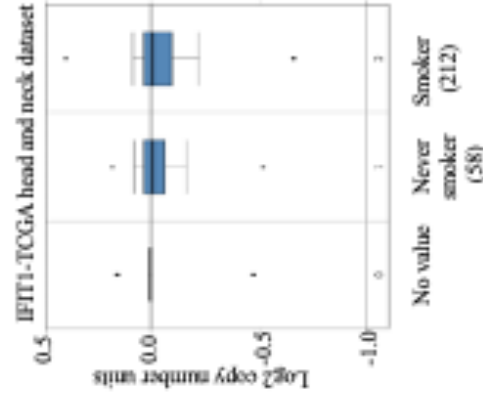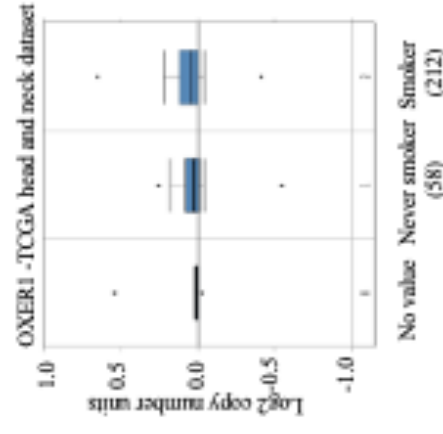

Supplement: Supplementary file 1 [file toxics-08-00073-s001.pdf]
